# Ketamine misuse: an update for primary care

**DOI:** 10.3399/bjgp23X731997

**Published:** 2023-01-27

**Authors:** Simon Gabriël Beerten, Catharina Matheï, Bert Aertgeerts

**Affiliations:** Department of Public Health and Primary Care, KU Leuven, Leuven.; Department of Public Health and Primary Care, KU Leuven, Leuven.; Department of Public Health and Primary Care, KU Leuven, Leuven.

## INTRODUCTION

Ketamine has long been known as a cheap and useful anaesthetic. Primarily owing to its dissociative properties, misuse of the drug became widespread in the 1990s.

The prevalence of ketamine misuse in England and Wales was at an all-time high in 2019 to 2020, at 0.8%.^1^ The younger age group (16 to 24 years of age) seems to be the most avid users: 3.2% of them used ketamine in that period. Although less prevalent, drug misuse in older adults should not be discounted.^2^ In general, much is still unknown about the epidemiology of ketamine, specifically regarding the frequency of its use and in what settings, and the trends over the years.

Physicians should be aware of ketamine misuse. The GP may be confronted with patients exhibiting signs and symptoms pointing to misuse and has an important role in screening for these patients, counselling them and referring them to specialised treatment if necessary.

## A BRIEF HISTORY

A derivative of phencyclidine (PCP), ketamine was initially used as a surgical anaesthetic, much like its precursor. It did have a better safety profile and tolerance, which contributed to its popularity, and can be used to achieve anaesthesia when clinical monitoring is not readily available, because respiration remains intact.^3^ It remains valuable both in human and veterinary medicine (as an anaesthetic), and has promising results in acute and chronic, as well as in paediatric post-operative pain management. In addition, it has potent anti-depressive, anti-suicidal, and even anti-inflammatory effects.^4^

Due to its properties, the potential for misuse of ketamine was already there from its inception and contributed to its diversion. Its use is well known in the rave and nightclub scene (‘Vitamin K’, ‘Special K’), because of its low price, short duration of action, and induction of powerful dissociative experiences. The first instances of misuse were signalled in the 1960s, with the 1990s seeing a global surge in ketamine’s popularity.^5^

## HOW DOES KETAMINE WORK?

Ketamine for recreational use is available as tablets or capsules containing a white powder and is usually snorted, or, less commonly, ingested orally.^3^ It is also available in liquid form for intramuscular or intravenous injection. Following ingestion, ketamine is rapidly distributed in the central nervous system (CNS) and its effects will last for up to 1.5 hours after insufflation. Its main route of metabolisation is hepatic, which is why the effect is much slower after oral ingestion (first pass effect). Common doses for single insufflation range from 100 to 200 mg.^3^

The CNS effects are mainly brought about by inhibition of the N-methyl-D-aspartate (NMDA) receptor, and, to a lesser degree, the catecholamine reuptake. This produces a psychosis-like state at subanaesthetic doses,^3^ and the reuptake inhibition is also responsible for the commonly observed increase in heart rate and blood pressure.

The main effects of an acute administration of ketamine are: anaesthesia, impaired motor function such as an ataxic gait, cognitive dissociation with disruption of space and time awareness, depersonalisation, and hallucinations at lower doses. Very high doses produce a state of profound detachment from reality, known as the ‘K-hole’. As with any NMDA-receptor antagonist, ketamine also negatively affects memory and cognition.^5^

## WHAT DOES ACUTE KETAMINE MISUSE LOOK LIKE?

As ketamine is often mixed with other drugs, such as cocaine and alcohol,^3^ the clinical manifestations of misuse are varied. In general, patients present with tachycardia and hypertension.

Patients with ketamine toxicity display a profound dissociation between various motor and higher cortical functions: they may appear drunk, unusually calm or agitated, or stuporous at very high doses. They may be disoriented or have auditory hallucinations. Gastrointestinal problems, such as abdominal pain and tenderness, nausea, and recurrent vomiting, might also be presentations of acute misuse.^6^ As fatalities are unlikely even in severe toxicity, deaths are usually caused by reckless behaviour such as jumping from heights or other misjudgements.^3^^,^^5^

Treatment of an acute overdose is largely second-line, but is mostly limited to supportive measures, including adequate pharmacological sedation if necessary.^7^

## WHAT ARE THE CONSEQUENCES OF LONG-TERM MISUSE?

Compared with non-users, deficits in long- and short-term memory have been reported, as well as depression.^5^

Over the last 10 years, case reports have surfaced regarding young patients with recurrent cystitis. Long-term ketamine misuse can indeed lead to a sterile ulcerative cystitis. Symptoms can resemble a bladder pain syndrome because of ketamine’s direct destructive effect on the urothelium: irritation, urgency, and frequency. A urine dipstick might show pyuria and haematuria, but no bacterial activity. Treatment is difficult, although cessation of ketamine use is always indicated.^8^

Gastrointestinal complaints are also common (‘K-cramps’). This usually presents as colicky upper abdominal pain, although the pain can be vague as well.^3^ While ulcerative cystitis can be the cause of lower abdominal pain, gastric and hepatic pathology (bile duct dilation) seem to cause the upper abdominal pain.^5^ There appears to be a dose–duration relationship with chronic complications.^5^^,^^9^

While ketamine does have addictive potential, there does not seem to be a clear withdrawal syndrome upon cessation.^3^ Some users do report intense cravings and physical symptoms when quitting. The addictive potential of ketamine is most likely twofold: the pleasurable, dissociative effects when taking the drug might reinforce its continued use, and the dopaminergic effects, while limited, act as an additional positive feedback loop. As with other drugs, use can be continued out of fear of withdrawal.

## WHAT IS THE GP’s ROLE IN ALL THIS?

GPs should be able to recognise the specific signs of acute and chronic ketamine misuse. In their management of patients, they should strive for complete abstinence. Of course, a ‘just stop’ message is unlikely to be productive and may be met with resistance and result in disengagement. Setting realistic goals and continuously evaluating the patient’s own motivation and goals prevent impatience, frustration, and disappointment.^10^

A good consultation begins with a thorough history-taking. It involves asking the patient about current or former drug use, as patients rarely bring this up themselves.^10^ Questions should then be specific: the GP should ask for misuse of cocaine, alcohol, or ketamine, for example. The general tone should be encouraging, non-judgemental, and understanding. Long-term ketamine misuse can lead to a myriad of symptoms, which can be difficult to put together without sufficient knowledge of the patient’s lifestyle.^5^

Young people presenting with depression, memory loss, or frequent urinary complaints should be screened for drug misuse, including ketamine, before delving into further diagnostic matters. Cystitis that seems resistant to antibiotics or with a persistently negative urinary culture, despite (sometimes visible) haematuria and pyuria, should prompt the GP to discuss ketamine misuse. This also avoids unnecessary antibiotic prescribing.^9^

The GP undoubtedly has an important role to play in encouraging patients to quit ketamine use and in supporting cessation with follow-up visits or referral to specialised centres (Box 1). However, given the relative scarcity of ketamine misuse, second-line expertise is likely to be limited.

**Box 1.** Red flags for referral

Acute ketamine toxicity. Usage of very high daily doses of ketamine. Profound or very specific memory deficits (unusual in ketamine misuse). Treatment-resistant cystitis; surprisingly negative urinary cultures despite presence of pyuria or haematuria. High social deprivation. Severe misuse of multiple drugs.

## SUMMARY

The dissociative anaesthetic ketamine has enjoyed widespread use in surgery. Its potential for recreational use was discovered soon, which led to a surge in misuse in the 1990s.

Usually snorted, the effects of ketamine are quick and short lasting. Recreational doses typically produce dissociative, hallucinatory sensations resembling psychosis. Heart rate and blood pressure increase. Its effects are mostly the direct result of NMDA-receptor inhibition, although it also influences catecholamine reuptake and dopamine activity.

Long-term effects are cognitive and memory deficits, ulcerative cystitis, and abdominal complaints. Cessation of use seems to be the only definitive therapy. The GP can play an important role in early recognition of misuse, early referral, and helping patients to quit. As misuse of ketamine continues to increase, the issue is unlikely to disappear in the future. Box 2 outlines this article’s key messages

**Box 2.** Key messages

Because of its low prevalence, ketamine misuse is not always visible in primary care. While GPs may only rarely be confronted with acute ketamine toxicity, they should be aware of the subtler signs of chronic misuse. A thorough history-taking, with special emphasis on ketamine misuse, is crucial in young patients presenting with depression, memory loss, or chronic urinary problems. Multidisciplinary management, with the GP performing a central, supporting role, is crucial in treating ketamine misuse.
